# Sepsis and COVID-19: outcomes in young adults in intensive care

**DOI:** 10.1590/0034-7167-2023-0037

**Published:** 2023-12-04

**Authors:** Talita Andrade Santos, José Edilson de Oliveira, Cassiane Dezoti da Fonseca, Dulce Aparecida Barbosa, Angélica Gonçalves da Silva Belasco, Carla Roberta Monteiro Miura

**Affiliations:** I Universidade Federal de São Paulo, São Paulo, São Paulo, Brazil

**Keywords:** Young Adult, Sepsis, Critical Care, Intensive Care Units, COVID-19, Adulto Joven, Sepsis, Cuidados Críticos, Unidades de Cuidados Intensivos, COVID-19, Adulto Jovem, Sepse, Cuidados Críticos, Unidade de Terapia Intensiva, COVID-19

## Abstract

**Objective:**

to verify sepsis incidence among young adults admitted to intensive care due to COVID-19 and to analyze its association with demographic, clinical and outcome variables.

**Methods:**

a quantitative, longitudinal, retrospective and analytical study, consisting of 58 adults aged 20 to 40 years in intensive care for SARS-CoV-2. It was carried out in a university hospital, from March 2020 to December 2021, with data collected from electronic medical records.

**Results:**

sepsis incidence was 65%. Sepsis was associated with acute kidney injury, use of vasoactive drugs and mechanical ventilation, being admitted to the emergency room, severity according to the Simplified Acute Physiology Score III and bacterial pulmonary co-infection, the latter being the most frequent etiology for sepsis.

**Conclusions:**

there was a high sepsis incidence, with 42% of deaths, which points to the importance of investing in preventive measures, especially in relation to bacterial pulmonary coinfections.

## RESUMO

**Objetivo:**

verificar a incidência de sepse entre adultos jovens admitidos na terapia intensiva por COVID-19 e analisar sua associação com variáveis demográficas, clínicas e desfechos.

**Métodos:**

estudo quantitativo, longitudinal, retrospectivo e analítico, composto por 58 adultos de 20 a 40 anos em terapia intensiva por SARS-CoV-2. Foi realizado em hospital universitário, no período de março de 2020 a dezembro de 2021, sendo os dados coletados dos prontuários eletrônicos.

**Resultados:**

a incidência de sepse foi de 65%. Estiveram associados à sepse lesão renal aguda, uso de droga vasoativa e de ventilação mecânica, ser admitido do pronto atendimento, a gravidade segundo o *Simplified Acute Physiology Score* III e a coinfecção pulmonar bacteriana, sendo essa última a etiologia mais frequente para sepse.

**Conclusões:**

observou-se alta incidência de sepse, com 42% de óbito, o que aponta para a importância de investimento em medidas preventivas, especialmente em relação às coinfecções pulmonares bacterianas.

## RESUMEN

**Objetivo:**

verificar la incidencia de sepsis entre adultos jóvenes ingresados en cuidados intensivos por COVID-19 y analizar su asociación con variables demográficas, clínicas y de resultado.

**Métodos:**

estudio cuantitativo, longitudinal, retrospectivo y analítico, formado por 58 adultos de 20 a 40 años en cuidados intensivos por SARS-CoV-2. Se llevó a cabo en un hospital universitario, de marzo de 2020 a diciembre de 2021, con datos recolectados de historias clínicas electrónicas.

**Resultados:**

la incidencia de sepsis fue del 65%. La lesión renal aguda, el uso de fármacos vasoactivos y ventilación mecánica, el ingreso a urgencias, la gravedad según el Simplified Acute Physiology Score III y la coinfección pulmonar bacteriana se asociaron a sepsis, siendo esta última la etiología más frecuente de sepsis.

**Conclusiones:**

hubo una alta incidencia de sepsis, con 42% de las muertes, lo que apunta a la importancia de invertir en medidas preventivas, especialmente en relación con las coinfecciones pulmonares bacterianas.

## INTRODUCTION

At the end of 2019, an outbreak of pneumonia of unknown origin occurred in Wuhan, China, which led to the discovery of a new type of coronavirus (SARS-CoV-2), with the coronavirus disease (COVID-19) being declared a pandemic by the World Health Organization on March 11, 2020^(1-2)^.

Systemic manifestations of coronavirus infection result from direct toxicity by the virus, endothelial and microvascular injury, with dysregulation of the immune system and stimulation of the hypercoagulable state, resulting in micro and macrovascular thrombosis. Effects on the respiratory system are more frequent and known, but virtually all other organ systems can be affected^(3)^.

Clinically, most patients infected with severe acute respiratory syndrome SARS-CoV-2 do not have severe symptoms, but almost 5% of patients have severe lung injury, requiring monitoring and intensive care, with mortality in Intensive Care Units (ICUs) can vary between 8% and 38% depending on the country^(4)^.

Sepsis is one of the leading causes of death in hospitals worldwide, with an estimated 11 million deaths every year. In Brazil, it is estimated that 400,000 cases occur among adults, of which 240,000 die^(5)^.

Sepsis is defined by the *Instituto Latino Americano de Sepse* (ILAS - Latin American Institute of Sepsis) as “a life-threatening organ dysfunction caused by a dysregulated host response to infection”^(6)^. The SPREAD study^(7)^, conducted by ILAS, pointed out that 30% of ICU beds in Brazil were occupied by patients with sepsis or septic shock. The lethality in these patients was 55%. Studies have pointed to multiple organ dysfunction, sepsis and septic shock as the main causes of death in patients with COVID-19^(8-9)^.

In addition to the problem of high mortality, sepsis represents a risk factor for the development of post-intensive care syndrome (PICS). PICS is defined as the set of new damage or worsening of previous conditions both in the physical, cognitive and psychological aspects that are established or progress after the course of a critical illness. The damage to the patient’s functionality can last for a few months or even years. The most common symptoms are weakness, the most common somatic alteration, occurring in more than 25% of survivors, in addition to loss of mobility, fatigue, anxious or depressed mood, sexual dysfunction, sleep disturbances, memory alterations, slowed thinking and difficulty of concentration^(10-11)^.

Moreover, patients who are victims of COVID-19, especially severe cases, may present the “post-acute COVID-19 syndrome” or “long covid”, which is defined as a combination of persistent signs and symptoms or late complications after 4 weeks. from the onset of the disease, where many patients experience fatigue, dyspnea, chest pain, cognitive changes, arthralgia and, therefore, a worsening of quality of life in the prolonged course of the disease^(12)^.

The young adult population, formed by adults between 20 and 40 years old, corresponds to 69.3% of the population, and is an economically active part of society^(13)^. Given the above, it is concerned that, in Brazil, at a given moment of the pandemic, there was a decrease in elderly people in cases of COVID-19 and an increase in younger adults in ICU beds, a phenomenon recognized as rejuvenation of the pandemic^(14)^.

Considering the above and the few reports of infectious complications in COVID-19 in this population, available so far, we believe that identifying the profile of young adults hospitalized with COVID-19 and the outcomes during their stay in intensive care could support better clinical practice multidisciplinary approach, aimed at the early identification and management of sepsis, with the aim of reducing morbidity and mortality and rehabilitation demands in this population.

## OBJECTIVE

To verify sepsis incidence among young adults admitted to the ICU due to a diagnosis of SARS-CoV-2 infection and its association with demographic, clinical and outcome data.

## METHODS

### Ethical aspects

The study took place in accordance with the recommendations of Resolution 466/2012, which deals with research on human beings, with the research project being approved by the Ethics Committee of the proposing institution, with waiver of signing the Informed Consent Form, as it uses secondary data.

### Study design, place and period

This is quantitative research, with a longitudinal, retrospective and analytical design, whose elaboration and description were carried out according to the assumptions recommended by STrengthening the Reporting of OBservational studies in Epidemiology (STROBE). The study setting was the ICUs specialized in the care of patients with COVID-19 of a university hospital that is a reference in teaching, research and assistance. The ICUs involved in the study had a total of 75 beds, with a total of 1,731 admissions. The data collection period was from March 2020 to December 2021.

### Population, sample, inclusion and exclusion criteria

For convenience, young adult patients (aged between 20 and 40 years) admitted to the ICUs, whose primary diagnosis was SARS-CoV-2 infection and who developed sepsis, were included. Patients whose electronic medical records were incomplete were excluded from the study.

### Study protocol

Demographic, clinical, and outcome data were collected using an electronic medical record after ethics committee approval, and the data were entered into the Research Electronic Data Capture (REDCap) data platform.

SARS-CoV-2 infection was confirmed by the positive result of the reverse transcriptase reaction test followed by polymerase chain reaction (RT-PCR).

Dependent variable was the occurrence of sepsis, which was screened considering the sepsis definition criteria established by ILAS^(15)^. The outcomes analyzed were discharge from the ICU or death during ICU stay.

The independent variables were classified into characteristics:

- Sociodemographic: age (years); sex (male/female); self-declared ethnicity (white/brown/black).
- Clinical: comorbidities (yes/no); length of stay (days); unit of origin (infirmary/emergency room); severity (SAPS III - Simplified Acute Physiology Score version III); use of mechanical ventilation (no/yes - time of use in days); vasoactive drug use (no/yes - time of use in days); delirium (yes/no); acute kidney injury (AKI) (no/yes - stratification); infectious focus.

Delirium incidence was verified by surveying the daily records of Confusion Assessment Method for the Intensive Care Unit (CAM-ICU)^(16)^, carried out in all shifts by nurses in the units studied. The AKI classification used was that of the Kidney Disease Improving Global Outcomes (KDIGO) guideline, which considers serum creatinine and urinary output values^(17)^.

### Analysis of results, and statistics

Data were analyzed according to the R program version 4.1.1. In the descriptive assessment, numerical variables were explored by means of minimum and maximum values, measures of centrality (mean) and dispersion (standard deviation), and categorical variables, explored by absolute frequencies and percentages. To assess categorical variables, the chi-square test or Fisher’s test was used. To test the difference between means, Student’s t test or non-parametric Mann-Whitney test were used. Statistical significance was considered for values of p≤0.05.

## RESULTS

During the studied period, we obtained 58 admissions of young adults with a primary diagnosis of SARS-CoV-2 infection, and the incidence rate of sepsis among them was 65.5% (n=38).

In the sample composition, 63.7% of young adults were male (n=37), with a mean age of 33 ± 5.62 years, with 81% (n=47) having pre-existing comorbidities, and 53.4% of them (n=31) were of white ethnicity. The origin of the Emergency Care Unit represented 63.7% of admissions (n=37).

As shown in Table 1, patients who developed sepsis during ICU stay had a mean age of 33 years, being mostly male (60.5%; n=23), and 50.0% of them (n=19) were of white ethnicity.

**Table 1:** Distribution of young adults diagnosed with SARS-CoV-2 (N = 58) admitted to the Intensive Care Unit according to demographic variables, São Paulo, São Paulo, Brazil, 2020

Variables	With sepsis (n= 38)	Without sepsis (n=20)	*p* value
Age in years			0.902^*^
Mean ± SD	33.63 ± 5.39	33.3. ± 5.60	
Minimum and maximum	22-40	23-40	
Sex, n (%)			0.475^#^
Male	23 (60.53)	14 (70.0)	
Female	15 (39.47)	6 (30.0)	
Ethnicity, n (%)			0.797 ^†^
White	19 (50.0)	12 (60.0)	
Brown	14 (36.84)	6 (30.0)	
Black	5 (13.16)	2 (10.0)	

* Mann-Whitney test;

# Chi-square test;

† Fisher’s exact test.

**Table 2:** Distribution of young adults diagnosed with SARS-CoV-2 (N = 58) admitted to the Intensive Care Unit according to clinical variables and outcome, São Paulo, São Paulo, Brazil, 2021

Variables	With sepsis (n= 38)	Without sepsis (n=20)	*p* value
SAPS III^≠^			0,008^*^
Minimum-maximum	29 - 74	24-61	
Unit of origin, n (%)			0.015^#^
Emergency Room	20 (52.63)	17(85.0)	
Inpatient unit	18 (47.37)	3 (15.0)	
Pre-existing comorbidities, n (%)			0.173 ^†^
Present	33 (86.84)	14 (70.0)	
Absent	5 (13.16)	6 (30.0)	
Delirium, n (%)			0.544 ^†^
Present	3 (7.89)	-	
Acute kidney injury, n (%)			**<0.001** ^†^
Present	27 (71. 05)	6 (40.0)	
KDIGO¥ 1	2 (5.26)	3 (15.0)	
KDIGO 2	2 (5.26)	2 (10.0)	
KDIGO 3	23 (60.53)	3 (15.0)	
Mechanical ventilation, n (%)			**<0.001^#^**
Yes	33 (86.34)	5 (25.0)	
No	5 (13.16)	15 (75.0)	
Vasoactive drug, n (%)			**<0.001^#^**
Yes	35 (92.11)	3 (15.0)	
No	3 (7.89)	17 (85.0)	
Focus of infection, n (%)			**<0.001** ^†^
Pulmonary	27 (71.05)	-	
Bloodstream	1(2.63)	-	
Pulmonary and bloodstream	8 (21.05)	-	
Others	2 (5.26)	-	
Outcome, n (%)			
ICU discharge	22 (57.89)	20 (100.0)	**<0.001^#^**
Death	16 (42.11)	-	

#Simplified Acute Physiology Score version III; ¥Kidney Disease: Improving Global Outcomes;

* Mann-Whitney test; #Chi-Square Test; †Fisher’s exact test; ICU - Intensive Care Unit.

Despite the high prevalence of pre-existing comorbidities among young people hospitalized for SARS-CoV-2 infection (86.8%), there was no statistically significant difference when analyzing the risk for developing sepsis. Among those who had sepsis, the most frequent comorbidities were cardiovascular (n=19), with emphasis on hypertension (n=16).

The unit of origin and the prediction of mortality on admission were associated with the development of sepsis (p=0.015 and p=0.008, respectively). Among the patients who developed sepsis, 52.6% of them (n=20) came from the Emergency Care Unit, and had a higher prediction of mortality on admission according to SAPS III.

Patients who had sepsis had an average length of stay (13.7 days) much longer than those who did not have this complication (7.9 days), reaching up to 75 days of stay in the ICU.

Despite a low delirium incidence among patients with sepsis (7.8%), this event was not present in patients without sepsis.

The use of mechanical ventilation was statistically significant (p<0.001) and seems to be linked to the most frequent infectious focus, the pulmonary focus (71.0%), since, among those who did not have sepsis, only 25.0% (n=5) used mechanical ventilation. Among young adults with pulmonary focus infection, 59.2% of them (n = 16) had bacterial co-infection, with two cases co-infection of two bacteria and one bacterial and fungal co-infection. The microorganisms found in samples of tracheal secretion cultures were *Klebsiella sp* (37.5%, n=6), *Staphylococcus sp* (31%, n=5), 3 *E. coli sp*+ *Staphylococcus sp* (18.75%, n= 3), *Serratia sp* (6.25%, n=1) and *Acinetobacter sp* and *Candida sp* (6.25%, n=1). The death rate among patients with pulmonary sepsis was 35.7%.

The vast majority of patients with sepsis (92.1%) required the use of vasoactive drugs, characterizing, therefore, evolution to septic shock. The vasoactive drug of choice was noradrenaline in 86.8% of cases.

As for AKI, 71.0% of patients with sepsis (n=27) developed AKI, 60.5% (n=23) in stage 3 of the KDIGO 3 classification, which is related to the use of renal replacement therapy by 57.8% (n=22) of patients with sepsis.

When analyzing the death outcome, it was observed that the death rate among patients with sepsis was 42.1% (n=16), and all patients who evolved without sepsis were discharged from the ICU (p<0.001).

## DISCUSSION

Young adults with COVID-19 hospitalized in intensive care had a sepsis rate of 65.5%, with pulmonary bacterial coinfection being the most frequent (59.2%). The emergence of secondary infections in the patient with COVID-19 can have many associated factors, including the dysregulated immune response, being a combination of immunosuppression induced by viruses and drugs^(18)^. It has been observed that SARS-CoV-2 infection can damage lung cells and infrastructure. Subsequently, the altered condition allows bacteria to increase adherence and invasion.

A study that analyzed predictors of secondary infection in patients hospitalized with COVID-19 showed that patients admitted to the ICU in the first 48 hours more frequently had a secondary infection compared to patients who were never admitted or admitted after 48 hours of admission. Other predictive factors were respiratory failure and severe lymphedema^(19)^.

Similar results are observed in a study carried out in New York involving 152 patients, whose pulmonary co-infection rate was 60.0%^(20)^. Another study involving 1,099 patients with COVID-19 with a mean age of 47 years also showed bacterial pneumonia as the main complication among patients with COVID-19^(21)^. A total of 86.43% of young adults involved in our study required mechanical ventilation and its use was associated with sepsis (p<0.001). A study involving mechanically ventilated patients with COVID-19 pointed to a 54% prevalence of ventilator-associated pneumonia (VAP)^(22)^. A study that identified more frequent microorganisms in cultures of tracheal aspirates associated with VAP in patients with COVID-19 isolated the following agents: *Acinetobacter sp, Pseudomonas aeruginosa*, yeast, *Klebsiella* and *Citrobacter sp* as well as another study^(18)^ that also found very similar results regarding the pathogens found in the subjects of the sample of the present study.

Although COVID-19 itself can cause acute respiratory decompensation, data on secondary bacterial pneumonia that play a role in this decompensation is limited. Evidence of need for support for secondary bacterial pneumonia includes one or more of the following: new or relapsing fever; new onset or change in character of sputum; new leukocytosis or new neutrophilia (or both); new relevant imaging findings; and new or increasing oxygen needs. It is also important to consider all other sources of nosocomial infections in these patients, such as indwelling central venous catheters or urinary tract catheters, and treat them accordingly^(22)^.

Among young adults with sepsis, 92.1% (n=35) used vasoactive drugs, characterizing the state of shock, a prevalence that differs from what it presented. A study^(23)^ that evaluated the frequency of hyperlactatemia in 68 patients with COVID-19 admitted to intensive care using vasopressors showed the development of septic shock, according to the Sepsis - 3 criteria, in only 1% of patients, inferring that the usual absence of hyperlactatemia in COVID-19 suggests that cellular/metabolic dysfunction is not a major contributor to organ dysfunction related to COVID-19. However, the cited study does not mention whether there are co-infections with COVID-19 infection.

AKI incidence among young adults was 56.8%. AKI is the second most common organ dysfunction in patients with COVID-19. Although the pathophysiology of AKI associated with COVID-19 is multifactorial, the virus has its mechanism of entry into cells through ACE2, expressed by renal cells^(18)^. AKI incidence among those who had sepsis was 71.0%, with the most frequent classification being KDIGO 3 (60.5%). There are several causes of AKI in the intensive care environment, and the process is usually multifactorial, with acute tubular necrosis as the most common mechanism, and sepsis and the use of nephrotoxic drugs for its treatment are important risk factors^(24)^.

The literature points out that both AKI and the use of antibiotics against co-infections have been associated with a poor prognosis for patients with COVID-19. It is recommended to have a high threshold for initiating additional antibiotics against a possible overlapping bacterial infection with COVID-19 due to the high prevalence of adverse renal effects^(25-26)^.

Zhou *et al*.^(27)^ found that, in the current COVID-19 pandemic, 50% of patients who died from COVID-19 had bacterial co-infections, a death rate very similar to that presented by young adults in the present study, which was 42.0%.

### Study limitations

The study has limitations because it was carried out in a single center, with a population characteristic of this service, and the number of participants does not characterize the general population, requiring larger studies with new participants. Another limitation found in this investigation is the collection of data from electronic medical records. Some data were missing or unavailable, thus excluding some research participants. The low availability of studies involving the young adult population with COVID-19 did not allow adequate comparison of findings.

### Contributions to nursing and health

The results found may encourage and add to new studies in the field of surveillance and care of young adult patients with SARS-CoV-2, which overlap this serious viral infection with one or more bacterial infections, generating a critical picture of difficult management and high mortality. It is believed that the data from this investigation will be able to direct the multidisciplinary clinical practice with a view to better outcomes.

## CONCLUSIONS

It is concluded that the development of sepsis in young adults admitted to the ICU occurred in 65% of cases, with pulmonary bacterial coinfection being the most frequent etiology, with a death rate of 42%. Being admitted from the Emergency Care Unit (p=0.015), with a high mortality prediction according to SAPS III (p=0.008), was associated with the development of sepsis. The development of AKI, the use of vasoactive drugs and the need for mechanical ventilation were also associated with sepsis in this population (p<0.001).
